# Work–Life Balance is an Illusion: Replace Guilt with Acceptance

**DOI:** 10.3389/fped.2015.00076

**Published:** 2015-09-17

**Authors:** Tessie W. October

**Affiliations:** ^1^Children’s National Health Systems, Washington, DC, USA

**Keywords:** parenting, burn out, pediatric, critical care, workload, stress, psychological

We walk with the ghosts of our patients every day of our lives. We chose critical care medicine because we love our work and are honored by the opportunity families give us to enter their lives when they are most vulnerable. We cannot develop these deep relationships that involve life and death then pretend those emotions and family stories do not enter our home lives. My work is my life and my life is my work. The concept of needing a balance between the two ignores that the fluid lines between work and life are frequently blurred.

When I see an ill child who shares a trait with one of my children, such as same age, hair color, smile, or sassiness, I have unintentionally allowed my life to enter my work realm. Similarly, while I am on vacation at the beach and subconsciously survey the scene to detect who looks ill and play out exactly how I would resuscitate that red lobster of a man, work has infiltrated my life.

I was riddled with grief and remorse while on maternity leave with my first child when I wrote and submitted my first NIH grant. I read articles on my smart phone while feeding. I did not nap when the baby napped as the mantra demands. I napped when I hit a writer’s block. I awoke at 02:00 a.m. while the baby was still asleep because I was motivated by an idea that entered my sensorium and I needed to sketch it out. I fielded questions from fellows seeking advice on their job search. I approved institutional review board submissions, phoned-in to research mentorship meetings, and submitted abstracts to national conferences. I also spent countless hours staring into the eyes of the child we created. When I returned to work, I was energized and felt rested, yet the constant chatter from my colleagues included statements judging my ability to have enjoyed my maternity leave. I must not have bonded with my child. How could my work–life balance be so off?

During my second maternity leave 2 years later, I made a promise to protect this valuable, precious time and to savor these magical moments. I disabled work email from my smart phone. I canceled all work-related meetings and slept. I did not submit any abstracts, manuscripts, or grants. The only articles I read were from the New York Times. My computer battery died unrecognized by me for weeks. I took that little girl everywhere and filled my day completely with child-centric activities. When I returned to work I was energized and rested. I was still immersed in guilt despite following the rules of maternity leave to a tee. This time guilt and overwhelming doom arose from the mountain of tasks ahead of me. It took three full days to manage emails and re-establish regularly scheduled meetings. There were no lectures scheduled, no new research protocols to activate and I was swamped by clinical commitments. I returned home exhausted with limited capacity to be an active parent. My work–life had swung completely into chaos. I asked again, how could my work–life balance be so off?

Two years later, I enjoyed the birth of my third child. I made no promises to myself. I put no restrictions on what I could and could not do while on maternity leave. I allowed myself to just be. I wrote when motivated, slept when tired, and parented when it seemed fitting. My days were fluid, unencumbered, productive, and loving. When I returned to work, I was energized and rested and guilt-free.

As intensivists, we make hundreds of decisions daily (1) and I would venture to guess that most decisions are good ones. Finding the key to work–life balance for me was having trust in my skills as an intensivist; acknowledging that these decision-making skills guide me in all my life decisions. I mostly make good decisions when deciding how best to spend my time. The most efficient and meaningful way to spend my time is to combine all aspects of my life and let them ebb and flow as naturally indicated.

As a woman in academic medicine, the burden to fulfill parental and work obligations is heightened. According to the Association of American Medical Colleges (AAMC), despite women comprising 47% of medical school classes, women make up 38% of full-time faculty, 21% full professors, and 15% of department chairs (2). This “leaky pipeline” adds an additional layer of external pressure put on women in academia to excel. It also can lead to a sense of isolation as women ascend the academic ladder often alone, even more so if she is an under-represented ethnic minority (3). The internal pressure to be an actively engaged mother can be equally crippling, especially in a field dominated by men who may not have the same pressures to perform at home. Even as women have increased their presence in the workforce, they continue to shoulder greater responsibilities at home (4).

I realized it was meaningless to separate work and life. Once we admit this reality to ourselves, we will relieve ourselves of the burden of fighting the internal battle (5). We will remove the guilt we feel when we check the clinical census while on vacation or create the kid’s activity schedule when we had committed to review that manuscript. We are successful physicians because we make good decisions and ultimately do what is needed. I am in balance because my work and life do not compete with each other, they are synergistic. Writing that grant while on my first maternity leave gave me the motivation and the drive to dive into work when I returned. Accomplishing something while I was supposed to hit the pause button on my career, made my life better. Spending uninterrupted time with my middle child is what I needed to know that quality, not quantity matters most to me. My family knows that when I am on clinical service they share mommy with other families. They also know that when I am off service they share mommy with research. My work colleagues know that when I take a mental health day, work commitments are marginalized. I frequently rely on my work skills as an intensivist to teach my children values of sharing, compassion, service, and responsibility. I also depend on my parenting skills of time management, negotiation, infusing laughter into everything I do, and the art of communication to improve the way I care for patients.

My work–life balance is not off. I am a better intensivist because of the joy my life brings to my work and I am a better parent because of the joy my work brings to my life. Every day when I come home to my children, especially after days when I was not able to give that gift to other parents, I appreciate my life more. Critical care medicine allows us to really, truly appreciate life as we often face death. This gift cannot be replicated in every profession. Critical care physicians should lead the field of medicine by shedding the burdens and embracing the reality of our work. I have accepted that the modern career physician does a disservice to the profession and to society by striving for the all-too-elusive idyllic work–life balance. It is time to redirect that energy into infusing life into one’s work. The next generation physician will have no other option than to be a clinician and researcher simultaneously. And of course, to prevent extinction, some of us will need to be parents too.

## Conflict of Interest Statement

The author declares that the research was conducted in the absence of any commercial or financial relationships that could be construed as a potential conflict of interest.
